# What's on the menu? A novel molecular gut content analysis to investigate the feeding behavior of phytophagous insects

**DOI:** 10.1002/ece3.70071

**Published:** 2024-09-24

**Authors:** Maja Fluch, Marta Chignola, Erika Corretto, Manfred Wolf, Stefanie Fischnaller, Luigimaria Borruso, Hannes Schuler

**Affiliations:** ^1^ Faculty of Agricultural, Environmental and Food Sciences Free University of Bozen‐Bolzano Bolzano‐Bozen Italy; ^2^ Competence Centre for Plant Health Free University of Bozen‐Bolzano Bolzano‐Bozen Italy; ^3^ Research Centre Laimburg Vadena Italy

**Keywords:** feeding behavior, food web, *Halyomorpha halys*, molecular gut content analysis, Nanopore Flongle, phytophagous insect

## Abstract

The relationship between phytophagous insects and plants is a central aspect of food webs and ecosystem functioning. The introduction of new species into an environment can have significant impacts on the food web of a native ecosystem. In many cases, there is a lack of knowledge on the biology and feeding behavior of invasive species prior their introduction and in the invaded regions. Gut content analyses of insects have provided valuable information on the host spectrum of insects. However, current approaches are time‐consuming and costly. Here, we describe a new molecular gut content analysis (GCA) approach using the Oxford Nanopore (ONT) Flongle sequencing platform to characterize the plant DNA present in the gut of the highly polyphagous insect species *Halyomorpha halys*. We demonstrate that this technique efficiently amplifies and correctly identifies plant DNA in a mock community. We performed a feeding experiment to determine the sensitivity of this approach and to assess how long the plant DNA can be detected. All plants used in the feeding experiment were correctly identified and detected after 56 days. Surprisingly, we also detected various plant genera that were not included in the feeding experiment and thus were likely ingested months before the experiment. Our study suggests that the GCA using the ONT Flongle sequencing platform represents a rapid and cost‐efficient diagnosis of the dietary preferences, host range, and the diversity of consumed plant species of pest insects with high precision.

## Graphical abstract

Schematic overview of the feeding experimental set up to investigate the suitability of the Oxford Nanopore Flongle device for the molecular gut content analysis of *Halyomorpha halys*.

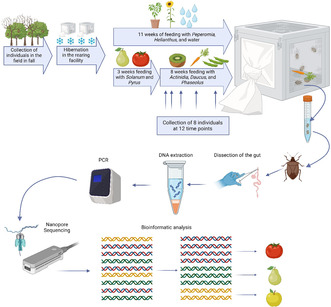

## 1 INTRODUCTION

Ecological networks describe the interactions of different organisms within ecosystems, including predation, parasitism, and herbivory (Ings et al., 2009; Ollivier et al., 2020). Food webs are seen as one part of these networks and describe the feeding relationships within a community (Ings et al., 2009). Analyzing different food‐webs helps to elucidate several questions, for example, the complexity of all interactions and the processes in ecosystems, co‐evolution, or the impact of biological invasions (Ollivier et al., 2020).

The introduction of invasive species into an ecosystem can have profound effects on the existing food webs, as they can feed on native plants or prey on native species (Kenis et al., 2009; Ollivier et al., 2020). In most of the cases, observational studies are used to assess the ecology of invasive species (Kenis et al., 2009). However, studying the feeding behavior based only on field collections has some limitations: field collections may be biased and might include false absences (Delmas et al., 2019). Moreover, field observations can describe the occurrence of an insect on a specific plant but can describe only to a limited extent whether a certain plant actually serves as feeding plant (Hereward & Walter, 2012; Kitson et al., 2013). Therefore, it is almost impossible to accurately reconstruct the exact feeding behavior just by observation (García‐Robledo et al., 2013; Zhu et al., 2019). Recently, molecular gut content analysis (GCA) was used to reconstruct the trophic interactions of insects by studying the contents of their digestive system to identify the ingested host DNA (Avanesyan et al., 2021; Eitzinger et al., 2019; Hayashi et al., 2020; Hepler et al., 2021; Wallinger et al., 2013; Zhu et al., 2019).

GCA has already been performed on a wide variety of organisms, from mammals to fishes to arthropods (Cooper et al., 2019; Fourie et al., 2022; Khanam et al., 2016; Macías‐Hernández et al., 2018; van der Reis et al., 2022). Previous studies focused on different research aims, such as the investigation of the persistence of DNA in the gut (Hepler et al., 2021; Macías‐Hernández et al., 2018), the identification of all host plants of a species (Barthel et al., 2020; Cooper et al., 2022; Hepler et al., 2023; Hereward & Walter, 2012; Pitt et al., 2024; Serrano et al., 2023), or the characterization of the feeding behavior across different life stages (Hepler et al., 2023). To reach these objectives, different combinations of DNA barcodes and sequencing platforms have been used. Most commonly plant DNA was identified using either the chloroplast genes *rbcL*, *trnF*, and *trnL*, the intergenic region *trnH‐psbA*, or the internal transcribed spacers (ITS) of nuclear ribosomal DNA (Barthel et al., 2020; García‐Robledo et al., 2013; Hepler et al., 2021; Staudacher et al., 2011; Wang et al., 2017). These genes were mainly sequenced with the classical Sanger sequencing approach (García‐Robledo et al., 2013; Hereward & Walter, 2012; Wang et al., 2017) or with the Illumina next‐generation sequencing (Eitzinger et al., 2019; Van Dijck et al., 2023). More recently, third‐generation sequencing platforms such as Pacific Biosciences (PacBio) and Oxford Nanopore Technologies (ONT) have been used for GCA (Cooper et al., 2019; Hepler et al., 2021; van der Reis et al., 2022). Although the ONT Flongle sequencing platform offers an ultrafast and cost‐efficient sequencing method, especially for amplicon sequencing (Avershina et al., 2022; Cha et al., 2023; Cuber et al., 2023), it has never been used to perform GCA on a herbivorous insect.

Here, we developed a novel GCA approach for phytophagous insects using the brown marmorated stink bug (BMSB) *Halyomorpha halys* (Stål) as study system. *H. halys* is an invasive insect in Europe and North America, originating from Northeast Asia (Cianferoni et al., 2018; Haye et al., 2014; Hoebeke & Carter, 2003; Leskey & Nielsen, 2018). It is considered highly polyphagous, with more than 300 described host plants (Kriticos et al., 2017). However, in most of the studies, the host plants are investigated by observational studies (Bergmann et al., 2016; Haye et al., 2014; Lee et al., 2013) which define the occurrence and/or the reproduction of *H. halys* on a specific plant, but do not give any information if they actually feed on the plant (Haye et al., 2014, 2015). For the validation of the GCA based on ONT Flongle sequencing, we (1) tested a mock community of plant DNA to assess the accuracy and efficiency of this platform and (2) set up a feeding experiment with alternating feeding plants to assess how long the DNA of the ingested plants is detectable.

## 2 METHODS

### 2.1 Collection and rearing of Halyomorpha halys

Individuals of *H. halys* were collected in October and November 2022 in Adige Valley, South Tyrol, Italy. Approximately 250 insects were kept at 9°C with a photoperiod of L8:D16 and 60% humidity to overwinter. After approximately 3 months, the temperature of the incubation chamber was increased to 18°C for 10 days with a photoperiod of L14:D10 and 60% humidity. The individuals were transferred to five 30 cm x 30 cm x 30 cm cages (BugDorm, MegaView Science Co., Ltd., Taiwan) with approximately 50 individuals per cage. The temperature was then increased to 25°C with a photoperiod of L16:D8 and 60% humidity. Four cages were used for the feeding experiment (feeding group) and one served as a control (control group), where only water and no food was provided.

**FIGURE 1:**
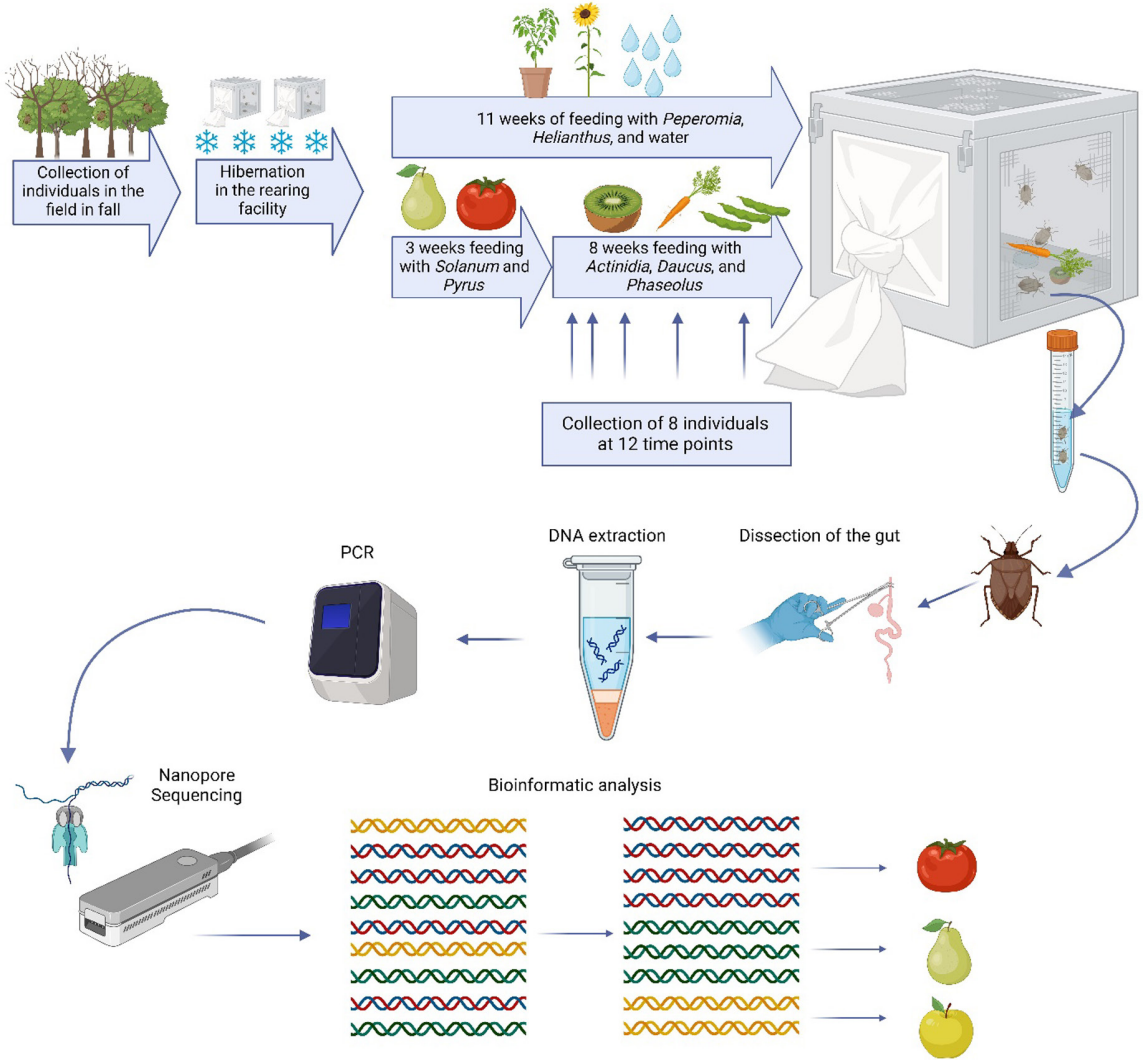
Schematic overview of the experimental setup. The feeding experiment and the collection of individuals was performed for 11 weeks. The insects were dissected, and the plant DNA was extracted. The ITS2 region was amplified via PCR and sequenced on the ONT Flongle flow cell. Sequences were quality‐filtered and taxonomic identification of the ingested plants was determined via BLAST. The figure was created with BioRender.com.

Each cage of the feeding group contained: paper towels serving as shelter for the bugs, cotton wool soaked with tap water, one plant of the genus *Peperomia*, sunflower seeds (*Helianthus annuus*), four to five cherry tomatoes (*Solanum lycopersicum*, variety: Axtar and Paskualeto), and one pear (*Pyrus communis*, variety: Abate Fetel). For 3 weeks, the bugs were provided with this diet ad libitum. To see how long the DNA of the first feeding plants can be detected, the diet was replaced with one carrot (*Daucus carota*) broken into three to four pieces, one kiwi (*Actinidia chinensis*), and around 25 green beans (*Phaseolus vulgaris*) while water, sunflower seeds, and peperomia were available in the cages for the duration of the experiment (Figure 1). The chosen feeding plants showed the best results in the rearing facility (Dingha & Jackai, 2017; Funayama, 2006; Medal et al., 2012; Taylor et al., 2017). Two times a week, the cages were cleaned by wiping all surfaces with 70% ethanol, paper towels, the cotton wool and, if needed, the feed was substituted. The bugs were collected at 12 time points during a period of 11 weeks (Table 1, Figure 1). The first collection (T0) was done 3 weeks after starting the experiment, before changing the diet (Figure 1). At each time point, two individuals were collected from each cage and directly stored in 100% ethanol at −20°C (Table 1). The control group was collected at five different time points (Table S1).

**TABLE 1:** Time points of *H. halys* individuals collected during the feeding experiment.

Time point	Days after changing the diet	Number of sequenced bugs (*n* = 99)	Average number of plant DNA reads per individual^a^	Average number of plant DNA reads after rarefaction^b^	Number of bugs with >1000 reads (*n* = 60)
T0	0	8	227 ± 230	0	0
T1	2	7	119 ± 96	0	0
T2	5	8	3933 ± 3957	5127 ± 3883	6
T3	7	8	3980 ± 2243	3980 ± 2243	7
T4	9	8	3238 ± 2490	4179 ± 2097	6
T5	12	5	4380 ± 5539	7133 ± 5739	3
T6	15	7	3653 ± 2294	4115 ± 2128	6
T7	20	7	2822 ± 1600	3174 ± 1426	6
T8	26	8	2181 ± 1580	2181 ± 1580	8
T9	34	8	2160 ± 2458	4282 ± 335	2
T10	44	6	0 ± 0	0	0
T11	56	7	3934 ± 2780	4435 ± 2676	6
Control	2–12	12	1737 ± 971	1964 ± 897	10

*Note*: The time points refer to the number of days after changing the diet after the 3 weeks post hibernation (Figure 1).

^a^ Average number of plant DNA reads per individual after filtering ± standard deviation. Individuals without reads assigned to plants were omitted.

^b^ Average number of plant DNA reads after rarefaction.

### 2.2 Dissection of the gut and DNA extraction

The gut of single individuals of *H. halys* was dissected using sterilized tweezers, scissors, and a needle. Despite careful dissection, some surrounding tissues might have been included in the material for DNA extraction. DNA was extracted using the Qiagen DNeasy Blood and Tissue Kit (QIAGEN GmbH, 40724 Hilden, Germany), following the manufacturer's instructions (Figure 1). The overview of individuals analyzed from the different time points is reported in Table 1.

### 2.3 Mock community composition

To validate if all the provided plants were correctly amplified, a mock community was prepared, consisting of the DNA extracted from peperomia leaves, sunflower seeds, and the flesh of pear, kiwi, bean, carrot, and tomato. DNA from individual plants was extracted using the Qiagen DNeasy Blood and Tissue Kit (QIAGEN GmbH, 40724 Hilden, Germany) following the manufacturer's instructions. The DNA concentration was measured with the Qubit 4 Fluorometer using the Qubit™ 1X dsDNA HS Assay Kit (ThermoFisher Scientific, Waltham, Massachusetts, USA). The DNA of all plants was pooled in equal amounts (10 ng each) and used for library preparation as described in the next paragraph. The mock community was sequenced in duplicates.

### 2.4 Library preparation and metabarcoding of the gut content

A PCR was performed using primers targeting the plant ITS2 region with Nanopore‐specific overhangs that were added at the 5′ end: ITS2_S2F (5′‐TTTCTGTTGGTGCTGATATTGC‐ATGCGATACTTGGTGTGAAT‐3′), modified from Chen et al. (2010) and ITS4 (R) (5′‐ACTTGCCTGTCGCTCTATCTTC‐TCCTCCGCTTATTGATATGC‐3′), modified from White et al. (1990), resulting in amplicons of approximately 400 bp. Each PCR reaction was set up in a laminar flow hood (GuardOne® Workstation‐Laminar Flow, Starlab (UK) LTD, Milton Keynes, United Kingdom) that was decontaminated using UV light for at least 30 min. Each reaction had a volume of 25 μL containing 2 μL of DNA, 1.25 μL of each primer (10 μM), 12.5 μL of Q5® Hot Start High‐Fidelity 2X Master Mix (New England Biolabs, Ipswich, Massachusetts, USA), and 8 μL of sterile water. The PCR was performed with the TurboCycler 2 Thermal Cycler (Blue‐Ray Biotech, Taipei City, Taiwan) under the following conditions: 98°C for 30 s, followed by 35 cycles of 98°C for 10 s, 51°C for 20 s, and 72°C for 25 s, with the final extension step at 72°C for 2 min. Each sample was run in duplicates and a negative control was added, where sterilized water instead of DNA was used. Amplification success was checked via electrophoresis on a 1% agarose gel. The positive duplicates were pooled, purified using the AMPure XP beads (Beckman Coulter Life Sciences, Indianapolis, USA), and used for the library preparation.

All samples were sequenced on the ONT Flongle flow cell (R9.4.1) using the PCR Barcoding Expansion 1–12 kit EXP‐PBC001 (Oxford Nanopore Technologies, Oxford, United Kingdom), which allowed the pooling of 12 individuals on a single flow cell. The barcoded samples were pooled in the same quantity of DNA (1.5 μg in total, 125 ng per sample) and the library was prepared with the Ligation Sequencing Kit SQK‐LSK110 (Oxford Nanopore Technologies, Oxford, United Kingdom) and the Flongle Sequencing Expansion kit EXP‐FSE001 (Oxford Nanopore Technologies, Oxford, United Kingdom) according to the manufacturer's instructions. Each sequencing was run for 18–48 h, until the number of passed reads (Phred Score ≥ 9) reached a plateau and a maximum of two pores were active.

### 2.5 Bioinformatic analysis

Demultiplexing and basecalling with a minimum Phred Score of 9 were performed during the run using the Guppy Basecaller (Guppy Basecalling Software, Oxford Nanopore Technologies plc. Version 6.5.7). The Guppy Barcoder was used to crop the barcodes. Sequences shorter than 250 bp and longer 600 bp were excluded from the analysis.

The sequences were then taxonomically assigned using MegaBLAST (Chen et al., 2015) and the NCBI database. To optimize and validate the method, different filtering settings were tested in R (R version 4.2.0) (R Core Team, 2022) on the sequences of the mock communities The percentage of nucleotide identity (pident) was tested in a range from 90% to 95% and the number of matching nucleotides (nident) was tested at 100, 150, and 200 bp, in combination with all the different pident (90, 91, 92, 93, 94, and 95%). The optimal filtering settings consisted of a pident ≥95% and a nident ≥200 bp. All sequences that did not match these requirements were excluded from the analysis. Only the BLAST hit with the highest pident was included in the analysis. The taxonomic assignment of the plants was performed at the genus level. The number of sequences for each genus was counted and all genera having less than 20 sequences were removed, together with all reads assigned to bacteria or fungi. To analyze the number of host plants per individual and the detection rates of the feeding plants across the different time points, we performed a rarefaction analysis using the vegan package in R to evaluate the amount of reads necessary to assess the whole plant diversity. All rarefaction curves showed a similar pattern reaching a plateau at 1000 reads (Figure S1). We therefore set a threshold of 1000 reads per individual where individuals with less than 1000 reads were excluded from the subsequent analysis (Table S2). Statistical analyses were performed in R using the packages car (Fox & Weisberg, 2019) and agricolae (de Mendiburu & Yaseen, 2020). Graphs were generated in R using the packages ggplot2 (Wickham, 2016) and cowplot (Wilke, 2023).

## 3 RESULTS

The sequencing of the two mock communities generated a total of 20,444 and 20,725 reads. After filtering, 7680 and 7897 reads were classified as plants. All seven plant genera provided in the feeding experiment were detected in both samples, but the number of reads assigned to each plant varied widely. Most reads were assigned to tomato (*Solanum*, 44.7% in sample A and 40.6% in sample B), green bean (*Phaseolus*, 32.4% and 32.7%), and sunflower (*Helianthus*, 16.4% and 16.7%), which accounts for a total of 93.5% and 90%. The remaining reads were assigned to carrot (*Daucus*, 2.9% and 4.7%), kiwi (*Actinidia*, 1.9% and 2.3%), pear (*Pyrus*, 0.3% and 1.5%), and *Peperomia* (0.6% and 0.7%) (Figure S2). The genus *Theobroma* was detected, although not added in the mock community (0.8% of reads in both samples). This might be the result of a contamination during the sample preparation. Two of the three negative controls included had zero reads assigned to plant DNA, while one sample had a low number of reads (87) assigned to *Daucus* and *Actinidia*.

By analyzing the gut content of 99 individuals across 12 different time points, we obtained in total from 2009 to 127,252 reads for each individual (mean number of reads: 42,907 ± 28,711). Overall, 4.26% ± 3.04% of these reads were assigned to bacteria, fungi, or were unclassified and were removed. Eleven individuals did not have any reads assigned to plants. To compare the number of host plants and the relative abundance of feeding hosts across time, we set a threshold of 1000 reads per individual based on the rarefaction curve, which reached a plateau at 1000 (Figure S1). In total, 39 individuals did not reach the threshold and were excluded from this analysis. All individuals from the time points T0, T1, and T10 were excluded from these analyses due to the low number of reads. Two to eight individuals (mean: 5.56 ± 1.88) remained for each of the other sampling time points (Table 1). The remaining 60 individuals from this dataset had between 1007 and 13,332 reads per individual (mean number of reads: 3709 ± 2559) (Table S2).

**FIGURE 2:**
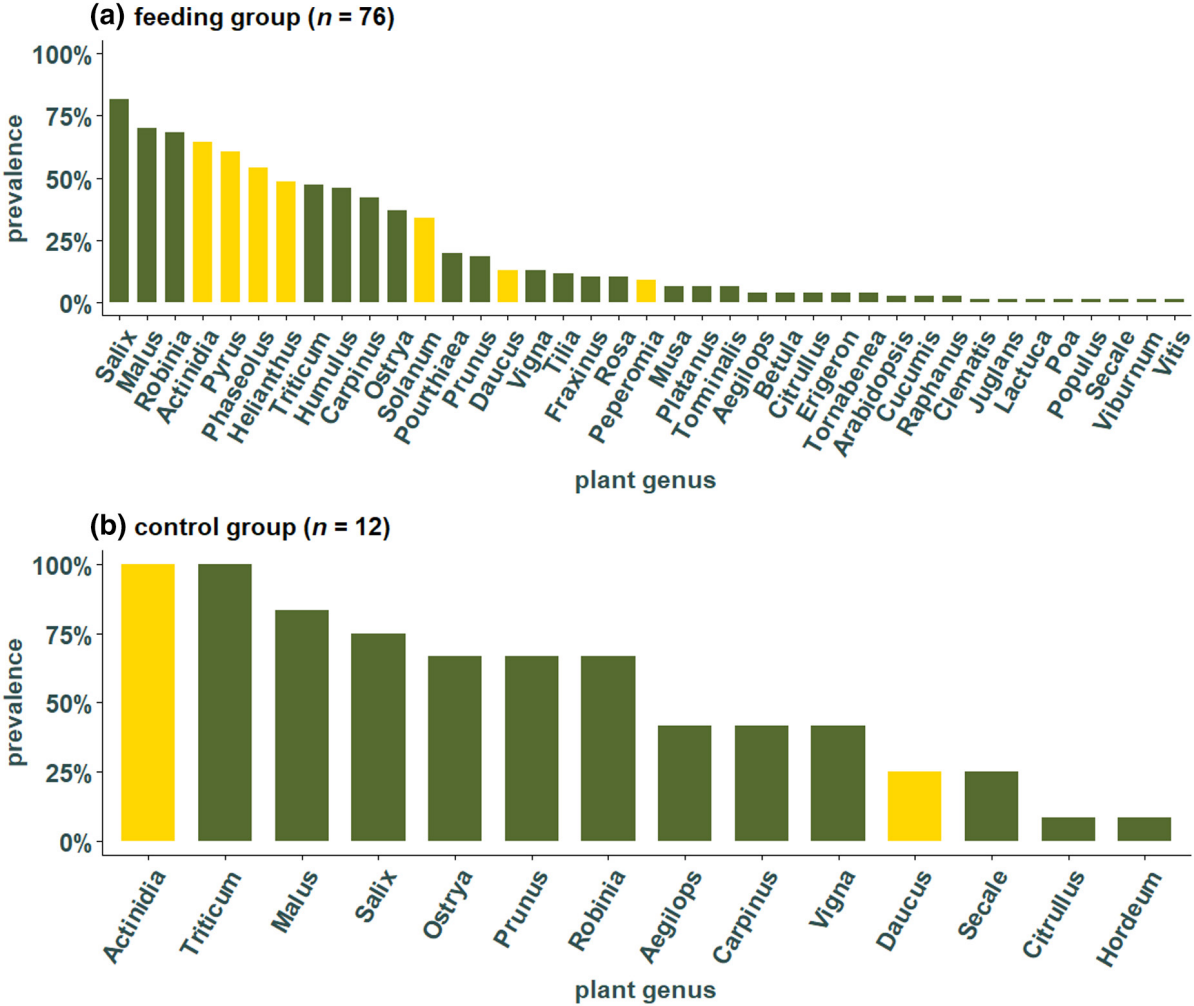
Prevalence of plants in the individuals of the feeding group (a) and in the control group (b). Data were not rarefied for this analysis. Yellow colored bars represent the seven plants provided during the feeding experiment: *Actinidia, Pyrus, Phaseolus, Helianthus, Solanum, Daucus*, and *Peperomia*; Green bars represent field plants which were detected but not included in the feeding experiment.

**FIGURE 3:**
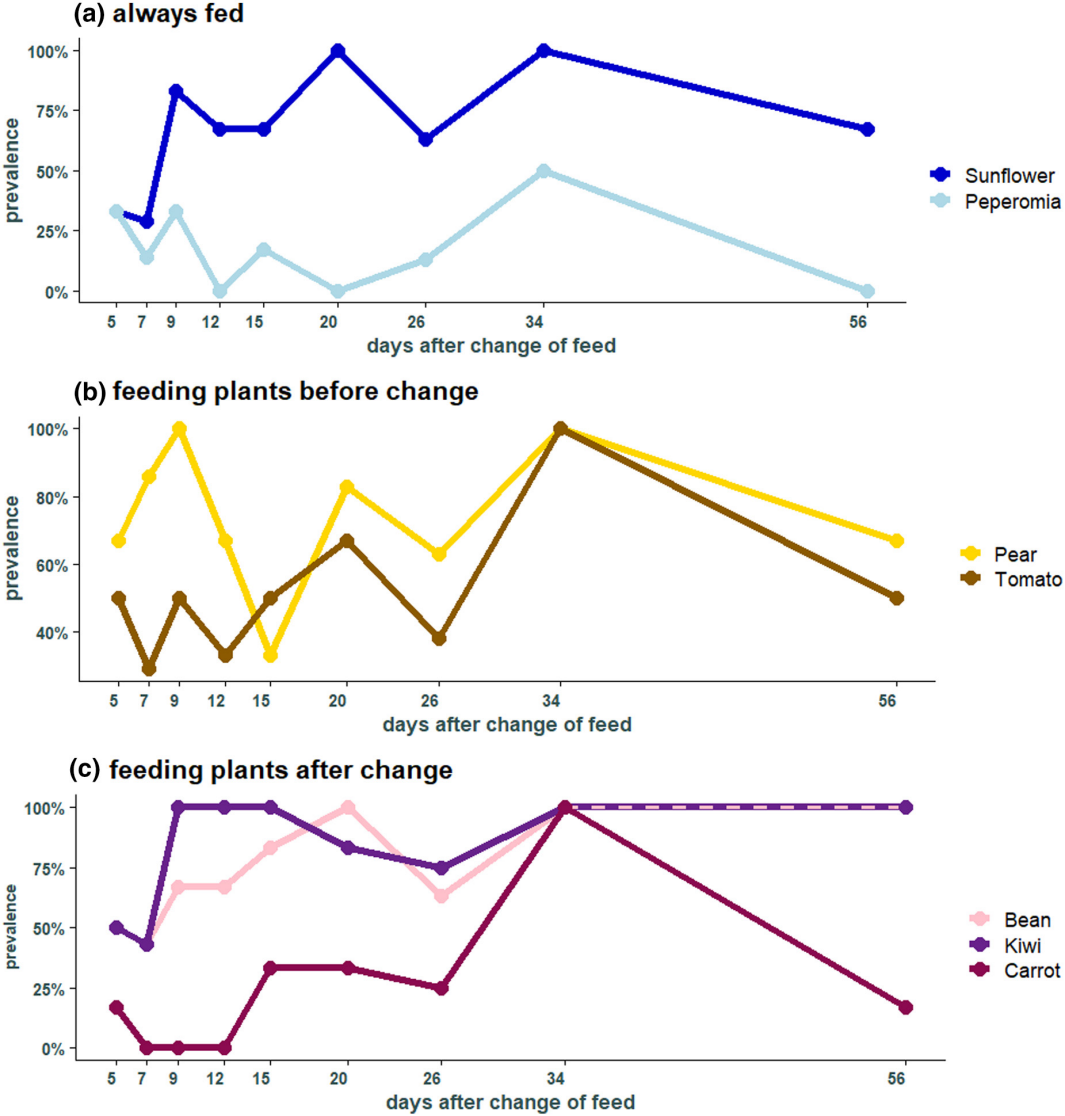
Prevalence of the different plant genera provided during the feeding experiment over the course of the 12 time points. (a) Plants that were fed across the whole experiment. (b) Plants that were fed in the first 3 weeks. (c) Plants that were fed from week three to the end of the experiment.

In the 76 individuals belonging to the feeding group, kiwi was found in most individuals (49 individuals), followed by pear (46), green beans (41), sunflower (37), tomato (26), carrot (10), and peperomia (7 individuals) (Figure 2a). Sunflower seeds, that were always present in the experimental cages, were found at each time point with a high prevalence, especially highly abundant after day 9 (Figure 3a). In contrast, *Peperomia*, which was also always provided, was not detected frequently, and was not detected in any individuals at days 12, 20, and 56 (Figure 3a). Pear and tomato that were fed only during the first 3 weeks after hibernation were both detected at each time point and pear was present in more individuals than tomato (Figure 3b). From the feeding plants that were provided in the second part of the experiment, only green beans and kiwi were found in high frequencies across the different collection times, both with a prevalence between 43% and 100%. In contrast, carrot was not present at three different times (day 7, 9, and 12) (Figure 3c).

Surprisingly, besides the seven feeding plants, additional 32 plant genera that were not included in the experiment were detected (Figure 2). The most frequent plant genera, *Salix*, *Malus*, and *Robinia* were found in a total of 62 (82%), 53 (70%), and 52 (68%) individuals of the feeding group and are and thus more prevalent than the most common feeding plants (Figure 2a). In the individuals of the control group, which were kept without food for around 4 months, a total of 14 different plant genera were detected in the gut of the 12 analyzed individuals. *Actinidia* and *Triticum* were found in all individuals and *Malus* was detected in 10 individuals, *Salix* in nine, *Ostrya*, *Prunus*, and *Robinia* were found in eight individuals (Figure 2b). Individuals from both the feeding and control group shared the following genera which were not provided in the feeding experiment: *Aegilops*, *Carpinus*, *Citrullus*, *Malus*, *Ostrya*, *Prunus*, *Robinia*, *Salix*, *Secale*, *Triticum*, and *Vigna*. Moreover, *Arabidopsis*, *Betula*, *Clematis*, *Cucumis*, *Erigeron*, *Fraxinus*, *Humulus*, *Juglans*, *Lactuca*, *Musa*, *Platanus*, *Poa*, *Populus*, *Pourthiaea*, *Raphanus*, *Rosa*, *Tilia*, *Torminalis*, *Viburnum*, and *Vitis* were found only in the feeding group (family and common names found in Table S3). The only genus that was detected in the control group but not the feeding group was *Hordeum*.

**FIGURE 4:**
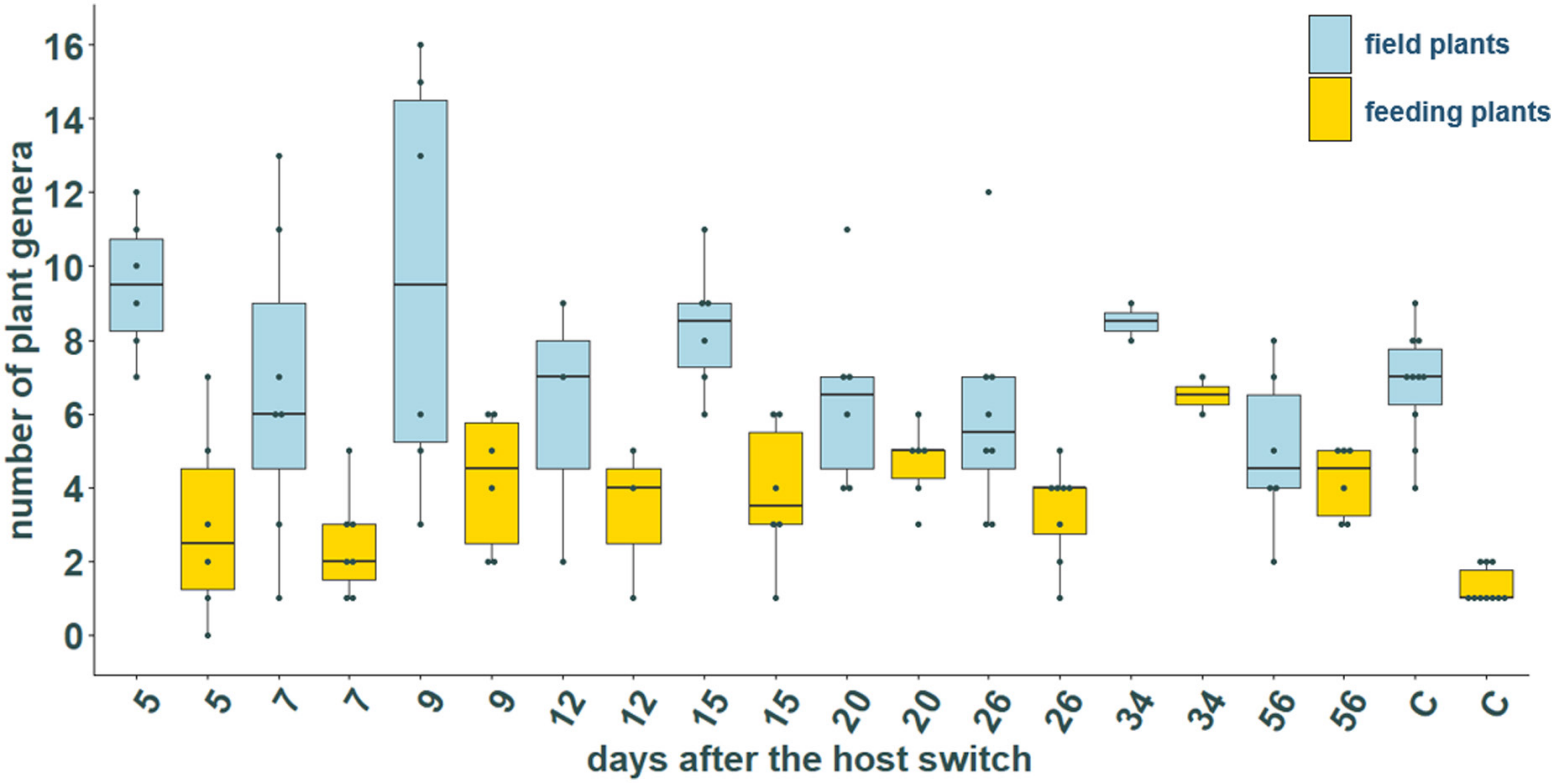
Number of plant genera found in the gut of *H. halys* at different time points. Blue – field plants: All plants that were not fed during the experiment. Yellow – feeding plants: The seven plants that were fed during the experiment. The numbers indicate the days after changing the diet after the 3 weeks post hibernation whereas C represent the number of plant genera found in the individuals belonging to the control group.

After excluding the 39 individuals which did not reach the threshold of 1000 reads, a mean of 11.02 ± 4.37 different plant genera have been detected in the feeding group. The lowest number of plant genera found in one individual was three, whereas one individual harbored DNA from 22 different plant genera. The control group had a significantly lower number of plant genera (mean 8.10 ± 1.45), with a minimum of five plants in one individual and a maximum of 10 plants in two individuals (Welch test, *p* < .05). No significant differences in the number of plants were detected between the different collection days (field plants: Kruskal–Wallis test, χ^2^ = 13.914, *p* > .05; feeding plants: Kruskal–Wallis test, χ^2^ = 25.927, *p* > .05; Figure 4). This indicates that the number of plant genera per individual remained constant throughout the feeding experiment.

## 4 DISCUSSION

Understanding the feeding behavior of animals is crucial to comprehend their biology and ecology. Especially polyphagous insect species can have a variety of food sources and disentangling the exact feeding hosts is tricky. Therefore, molecular gut content analysis is a powerful tool to assess the complete diet of an organism. A common limitation of this approach is that the sensitivity is often unknown and recent studies have shown that the ingested DNA can be traced back only by a limited time, that is, a few hours or days (Briem et al., 2018; Hepler et al., 2021; Pumariño et al., 2011). Here, we present a novel method for DNA metabarcoding using the ONT Flongle device. The approach has the advantage to provide fast results, as sequencing can be performed instantly in the laboratory. Moreover, the method is cheap, as multiple samples can be barcoded and sequenced in parallel on one Flongle flow cell. To test its sensitiveness, we set up a feeding experiment to assess the time frame within which the DNA of the ingested plants can be detected.

Previous studies showed that the post‐feeding detection of plant DNA ranges according to the feeding behavior of the insect (Briem et al., 2018). In case of chewing insects, the DNA of the ingested plant was found after more than 24 h in lepidopteran larvae (Pumariño et al., 2011) and easily up to 72 h in the larvae of click beetles (Staudacher et al., 2011), while the post‐feeding DNA detection decreased significantly after 16–20 h in mirid bugs (Wang et al., 2017). Briem et al. (2018) speculated, that the plant DNA from liquids ingested by piercing‐sucking bugs compared to DNA in tissues absorbed by chewing insects might be more easily degraded and therefore have a shorter post‐feeding detection rate (Briem et al., 2018). This was challenged by Cooper et al. (2019), who investigated the feeding hosts of the phloem‐feeding psyllids and detected plant DNA weeks or months after feeding (Cooper et al., 2019).

To our knowledge, only two studies performed a GCA of Pentatomidae. Fourie et al. (2022) showed a variety of host plants of the two‐spotted stink bug *Bathycoelia distincta* in the wild, whereas Hepler et al. (2021) investigated the plant DNA of laboratory‐fed individuals of *H. halys* and detected plant DNA 3–14 days after ingestion, depending on the used marker. To our surprise, in our study, we did not only find the plants initially fed even after 56 days, but we also found an unexpectedly high diversity of plant genera which were not included in the feeding experiment. They have likely been ingested before our collection and thus before the winter diapause, months before the start of the feeding experiment. An explanation for the long persistence of DNA in the gut of *H. halys* can be found in the anatomy and physiology of plant‐sucking heteropterans. If the diet of plant‐sucking heteropteran's does not or only in small amounts include solid waste material, there is a closure between the different parts of the midgut (Chapman, 1998). The midgut is divided into the regions M1, M2, M3 (in some species M4B), and M4. The latter is the main region where symbionts are found and in between M3 and M4 a narrow region (called “constricted region”) was described in several stink bug species. This narrow part hinders the food fluid to proceed to the M4 region, which is then excreted via the Malpighian tubes and in the feces (Ohbayashi et al., 2015). The midgut of the southern green stink bug *Nezara viridula* (Hemiptera: Pentatomidae) blocks the ingested material between M3 and M4 (Lomate & Bonning, 2016). This is causing an accumulation of the food in the M3 region (Cantón & Bonning, 2019). Additionally, the activity of nucleases in the midgut of *H. halys* and *N. viridula* is very low compared to the saliva and salivary glands (Cantón & Bonning, 2019; Lomate & Bonning, 2016, 2018). We therefore speculate that the ingested food is accumulated in the gut of *H. halys*, where there are no or only very few nucleases present to degrade the plant DNA.

The long persistence of DNA in the gut of *H. halys* also explains, why we cannot see any significant differences in the number of detected plants found on the different collection days and after switching the feeding plants during the feeding experiment. In total, we detected 32 plant genera that were not fed in the experiment. All plants detected in this work are present in the study region (South Tyrol, Italy). Some of them (*Carpinus*, *Malus*, *Prunus*, *Salix*, *Triticum*, *Fraxinus*, *Humulus*, *Juglans*, *Platanus*, *Rosa*, *Viburnum*, *Vitis*, *Robinia*, *Populus*, *Tilia*, *Secale*, *Betula*, and *Vigna*) were already described as hosts or associated plants of *H. halys* (Bakken et al., 2015; Bergmann et al., 2016; Bosco et al., 2020; Dingha et al., 2021; Haye et al., 2014, 2015; Hess et al., 2022; Holthouse et al., 2021; Lee et al., 2013; Maistrello et al., 2016; Rice et al., 2014; Wermelinger et al., 2008). Remarkably, a total of 13 associated plants were, to the best of our knowledge, not described in literature yet: *Aegilops*, *Citrullus*, *Ostrya*, *Arabidopsis*, *Clematis*, *Erigeron*, *Lactuca*, *Musa*, *Poa*, *Photinia*, *Raphanus*, *Torminalis*, and *Hordeum*. Thus, by assessing only a small number of individuals (*n* = 88), we were able to identify 13 new host genera. Whether these genera represent an additional reproductive host or only an occasional host plant, needs to be confirmed in future studies.

While our results highlight the potential of our newly developed approach, this method still has some limitations. Although the ONT technology allows a rapid, cost‐efficient, high‐throughput sequencing approach, it generally generates sequences at lower quality with relatively high error rates (Loit et al., 2019; Srivathsan et al., 2018; Tedersoo et al., 2019). The recently released R10.4.1 flow cell is expected to provide a higher quality of the produced reads due to the improved accuracy (Szoboszlay et al., 2023). By using the R9.4 Flongle flow cells, the low quality of our reads constrained us to a stringent filtering and a taxonomic determination to the genus level. We expect the new technology to allow a higher sequencing output and a quality that should allow to detect plants up to the species level. The results of the mock communities, where the amplification of some plants is up to 140‐fold higher than for others, even if present at the same concentration, indicates that the sequencing output cannot be interpreted quantitatively. Therefore, our approach cannot provide any quantitative information about the amount of plant DNA of each genus. The most plausible explanation for this might be due to a difference in the amplification efficiency of the primers for the different plant taxa (Hepler et al., 2021; Zhu et al., 2019). Additionally, the plant ITS region is generally present in multiple copies in the plant genome (Cheng et al., 2016), thus explaining the varying amplification efficiencies of different plant species having variable numbers of copies. Hence, employing additional plant‐specific markers should provide also quantitative results. Nevertheless, our study demonstrated that the ONT Flongle device is a suitable instrument for conducting GCA quickly and cost‐efficiently. It enables us to evaluate the feeding habits of phytophagous insects and can potentially be applied to other food webs.

## AUTHOR CONTRIBUTIONS

**Maja Fluch:** Conceptualization (equal); data curation (lead); formal analysis (lead); investigation (lead); methodology (lead); validation (lead); visualization (lead); writing – original draft (lead). **Marta Chignola:** Formal analysis (supporting); investigation (equal). **Erika Corretto:** Conceptualization (supporting); investigation (supporting); methodology (supporting); supervision (supporting); writing – review and editing (supporting). **Manfred Wolf:** Conceptualization (supporting); methodology (supporting). **Stefanie Fischnaller:** Conceptualization (supporting); investigation (supporting); methodology (supporting). **Luigimaria Borruso:** Formal analysis (supporting); methodology (supporting); software (supporting). **Hannes Schuler:** Conceptualization (lead); funding acquisition (lead); methodology (equal); project administration (lead); resources (lead); supervision (lead); validation (supporting); writing – original draft (supporting).

## CONFLICT OF INTEREST STATEMENT

There are no conflicts of interests.

## Supporting information


Appendix S1.

## Data Availability

All raw data and metadata are available at the NCBI GenBank under the accession number PRJNA1126037.
