# Supplementary material for: What's on the menu? A novel molecular gut content analysis to investigate the feeding behavior of phytophagous insects
Source: Ecol Evol. 2024 Sep 24;14(9):e70071. doi: 10.1002/ece3.70071 (PMC11421882; doi:10.1002/ece3.70071)
Supplement: Supplementary file 1 — Appendix S1. [file ECE3-14-e70071-s001.zip › Supplemental_Information.docx]

**Supplemental Information for:**

**What’s on the menu? A novel molecular gut content analysis to investigate the feeding behavior of phytophagous insects**

Maja Fluch, Marta Chignola, Erika Corretto, Manfred Wolf, Stefanie Fischnaller, Luigimaria Borruso, Hannes Schuler

**Figure S1** The rarefaction curves generated using the vegan package in R. All of them show a plateau at 1,000 reads. Therefore, the minimum number of reads needed for the sample to be included in the analysis was set to 1,000.


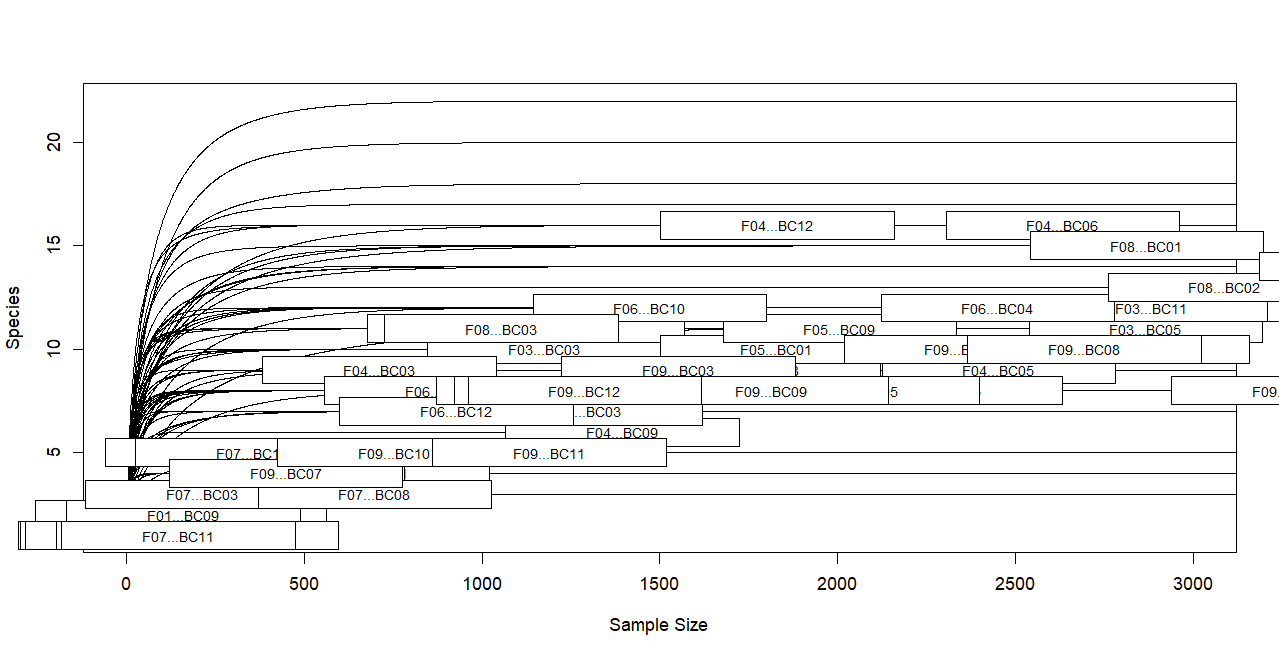


**Figure S2** The relative abundance of the plant genera detected in the mock communities.


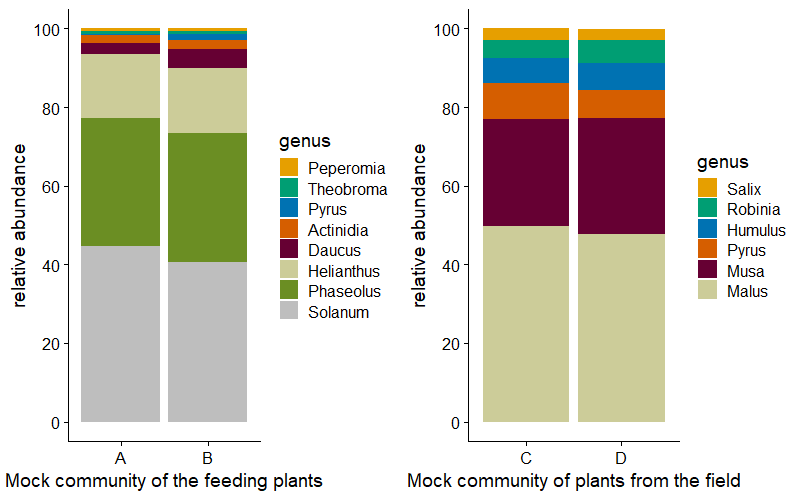


**Table S1** The number of individuals belonging to the control group that were sequenced.

| Time point | Days after changing the diet | Number of individuals (alive) | Number of individuals (dead) |
| --- | --- | --- | --- |
| T1 | 2 | 5 | 0 |
| T2 | 5 | 2 | 2 |
| T3 | 7 | 2 | 0 |
| T5 | 12 | 1 | 0 |
